# The use of a cognitive aid app supports guideline-conforming cardiopulmonary resuscitations: A randomized study in a high-fidelity simulation

**DOI:** 10.1016/j.resplu.2021.100152

**Published:** 2021-08-17

**Authors:** T. Grundgeiger, F. Hahn, T. Wurmb, P. Meybohm, O. Happel

**Affiliations:** a Institute Human-Computer-Media, Julius-Maximilians-Universität Würzburg, Oswald-Külpe-Weg 82, 97074 Würzburg, Germany; b Department of Anaesthesiology, Intensive Care, Emergency and Pain Medicine, University Hospital of Würzburg, Oberdürrbacher Straße 6, 97080 Würzburg, Germany

**Keywords:** Cardiopulmonary resuscitation, Checklists, Crisis management, Information technology, Medical emergency team, Simulation

## Abstract

**Aim:**

Cardiac arrests require fast, well-timed, and well-coordinated interventions delivered by several staff members. We evaluated a cognitive aid that works as an attentional aid to support specifically the timing and coordination of these interventions. We report the results of an experimental, simulation-based evaluation of the tablet-based cognitive aid in performing guideline-conforming cardiopulmonary resuscitation.

**Methods:**

In a parallel group design, emergency teams (one qualified emergency physician as team leader and one qualified nurse) were randomly assigned to the cognitive aid application (CA App) group or the no application (No App) group and then participated in a simulated scenario of a cardiac arrest. The primary outcome was a cardiopulmonary resuscitation performance score ranging from zero to two for each team based on the videotaped scenarios in relation to twelve performance variables derived from the European Resuscitation Guidelines. As a secondary outcome, we measured the participants’ subjective workload.

**Results:**

A total of 67 teams participated. The CA App group (n = 32 teams) showed significantly better cardiopulmonary resuscitation performance than the No App group (n = 31 teams; mean difference = 0.23, 95 %CI = 0.08 to 0.38, p = 0.002, d = 0.83). The CA App group team leaders indicated significantly less mental and physical demand and less effort to achieve their performance compared to the No App group team leaders.

**Conclusions:**

Among well-trained in-hospital emergency teams, the cognitive aid could improve cardiopulmonary resuscitation coordination performance and decrease mental workload.

## Introduction

High-quality cardiopulmonary resuscitation (CPR) is critical for patient outcome.^1^ However, even for well-trained staff of in-hospital emergency teams, coordinating CPR actions according to guidelines is challenging.2., 3. Possible challenges are reduced cognitive functions due to stress,^4^ distorted time perception,^5^ or the challenges of attending to and coordinating several threads of actions of CPR and other tasks such as asking for patient information.6., 7.

**Fig. 1:**
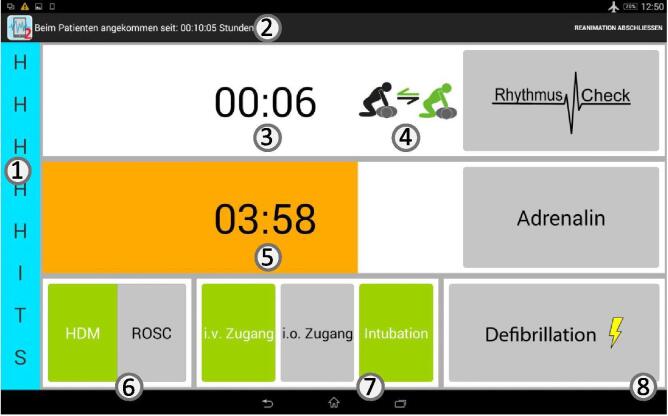
Description of cognitive aid application. Screenshot of tablet-based application‘s main screen: (1) German version of the Hs and Ts mnemonic (extends if touched and blue background flickers after each rhythm check), (2) time since arrival at patient, (3) time since last heart rhythm check (and button to document a check on the far right labeled “Rhythmus Check”; documentation will reset timer; timer background will turn orange/red at 01:40/2:00 and the tablet vibrates), (4) icon to remind user to exchange person doing chest compressions (the icon appears when the rhythm check timer approaches 01:40), (5) time since last adrenalin administration (in the screenshot, the orange color indicates that next administration should be considered), (6) toggle button to document chest compressions (“HDM”) or return to spontaneous circulation (“ROSC”), (7) three buttons to document specific medical interventions, (8) button to document defibrillation (after every third defibrillation, a syringe icon appears to consider amiodarone administration).

Research has shown that cognitive aids — artifacts that support a user during the actual performance of a task — can provide support in various crisis situations8., 9., 10., 11., 12., 13. and foster patient safety.^14^ In a multi-year project, we developed a cognitive aid tablet application to specifically support well-trained in-hospital emergency teams.15., 16., 17. During the user-centred design process, it became apparent that these teams need an attentional aid that supports the timing and coordination of events and that the need for a memory aid that prompts steps or guides the team leader through an algorithm was less pronounced. In addition, the literature showed that wrong event timing of, for example, defibrillations contributes to worse patient outcomes.^3^ We focused on supporting the timing and coordination of actions and showed that the application could improve the non-technical performance of emergency teams.^15^ The application provides several timers (e.g., for 2-min heart rhythm analysis cycle) with different forms of alerts (prepare task, do task). A description of the application can be found in Fig. 1.

To evaluate whether the application can improve the coordination of guideline-conforming CPR, we conducted a randomized study using a simulated cardiac arrest scenario. A CPR performance score based on the mean of the twelve CPR-related variables was defined as primary outcome. We expected that a cognitive aid application group would show a higher CPR performance score compared to a no application group.

## Methods

In a parallel group design, teams were blinded to the aim of the study and assigned to the groups (CA App vs. No App) by drawing a token out of an urn. The data were collected at two local hospitals. All physicians had obtained the German certificate for prehospital emergency medicine and had at least two years of training in the field of acute care medicine. All nurses had an acute care working background. The local university ethics committee approved the study (40/17-ge). Written informed consent was obtained from all participants.

Participants in the CA App group received a three-minute training to familiarize themselves with the application. Physicians were informed about the app’s functions and were instructed to hold the tablet in their hands and use the app’s functions as much as possible in the scenario. All teams waited in the hallway outside of the simulation room until a member of the simulation team called and informed the team about the emergency. Subsequently, a scenario of a witnessed in-hospital collapse due to cardiac arrest (see supplementary material) was performed and video recorded. The manikin was a *Resusci Anne*® simulator (Laerdal, Stavanger, Norway). Finally, physicians and nurses answered a demographic questionnaire and the NASA TLX^18^ without the weighting of the single subscales (the so-called TLX raw19., 20.). The TLX raw served as a secondary outcome measure.

In contrast to previous studies,8., 9., 11. we expected that these teams would perform all CPR actions and therefore measured twelve objectively assessable CPR performance variables and scored the performance quality instead of scoring for conducted or not conducted actions. We combined these variables to a CPR performance score which was the primary outcome measure in this study.

We defined no-flow time as the time of cardiac arrest during which no chest compressions were being performed. The no-flow fraction is the ratio between no-flow time and the total time of cardiac arrest. The no-flow fraction was calculated based on a manual analysis of the video recordings. We also analysed the average chest compression depth and rate based on the data recorded by the software of the simulator (*SimPad*®, Laerdal, Stavanger, Norway). Finally, time to first heart rhythm analysis and defibrillation were defined as actual time of first analysis/defibrillation minus arrival time at the patient.

To investigate guideline-conforming heart rhythm check intervals and changes of the person providing chest compressions, we coded the start and finish time of each of these events and subtracted this interval by two minutes. If the result was zero or negative, we considered this as a guideline^20^ conform change with a delay of zero. Otherwise, we noted the delay in seconds. Based on the current guidelines, the resulting averages of these values were considered as deviation from the guidelines.^20^

To investigate guideline-conforming adrenaline (epinephrine) administration, we coded adrenaline administration starting with the first instance. We considered any differences less than three minutes (i.e., administration too early) or more than five minutes (i.e., administration too late) as deviations from the guidelines.^20^ Again, we averaged the guideline conform administrations (i.e., within three to five minutes intervals and therefore zero delay) and delays for the CPR performance score calculation.

We also considered the expected number of changes of the person providing chest compressions, heart rhythm checks, and adrenaline administration based on the scenario length (i.e., longer scenarios required more actions; see Supplemental Table 1 for scoring). Finally, we analyzed whether amiodarone was administered.

**Table 1:** Demographic data and scenario length separated for the cognitive aid application (CA App) and the no application (No App) groups. Data are presented as frequencies or median (IQR). Statistical test are Fisher exact test and Mann-Whitney-U tests and effect sizes are rank biserial correlations.

Variable		CA App (n = 32 teams)	No App (n = 31 teams)	p value and effect size
Female/male gender	Physician	8/24	10/21	p = 0.585
	Nurse	21/11	20/11	p = 1

Age (years)	Physician	37.0 (8.0)	34.5 (8.0)	p = 0.250, r = 0.168
	Nurse	30.5 (13)	32 (14)	p = 0.210, r = -0.183

Work experience (years)	Physician	5.5 (5.0)	8.0 (7.0)	p = 0.152, r = -0.211
	Nurse	8 (6.4)	9.0 (19.1)	p = 0.512, r = 0.097

Scenario length (mm:ss)		15:43 (03:17)	15:02 (02:27)	p = 0.163, r = 0.205

The variables were either recorded by the *SimPad*® software (chest compression depth and rate) or analysed by a single person. To assess reliability, a second person coded 10% of the data (i.e., three randomly chosen teams from each group). We calculated an interclass correlation for the two coders based on the no-flow time, time to first rhythm check, time to first shock, the deviation of changing the person providing chest compressions, heart rhythm analysis, and adrenalin administration of each team (i.e., 6 × 6 data points). The interclass correlation showed an excellent agreement of 0.923 (95% CI: 0.855–0.960; single rater, absolute-agreement, 2-way mixed-effects model).^21^

Based on guidelines,^20^ we scored 0, 1, or 2 points for each variable depending on the quality of the performance (Table 2). As in previous work,^15^ we added a 15% margin to separate between 0, 1, and 2 points. For example, time to first shock scored 2 points if the time was ≤120 s, 1 point if the time was between 121–138 s, and 0 points if the time was >138 s. Note that the result pattern did not change when we did not use a margin and transformed all scores of 1 to scores of 0. Because all variables are part of the current guidelines, we calculated a CPR performance score for each team by averaging the scores of the twelve variables.

**Table 2:** Description and results of cardiopulmonary resuscitation performance scoring. The percentages indicate the percentage of a score of 1, 2, or 3 in each group (no application = No App; cognitive aid application = CA App). The means (SD) indicate the average value for each variable in each group. For the expected number of person providing chest compression changes, adrenaline administration, and heart rhythm checks scoring please refer to Supplemental Table 1.

Variable			No App	CA App
No-flow fraction (%)	Score 0	>20	9.7%	9.4%
	Score 1	20–15	25.8%	12.5%
	Score 2	<=15	64.5%	78.1%
	M (SD)		14.06 (4.40)	13.28 (4.09)

Average chest compression depth (cm)+	Score 0	other	72.7%	53.8%
	Score 1	-	-	-
	Score 2	5–6	27.3%	46.2%
	M (SD)		4.0 (1.0)	4.6 (1.0)

Average chest compression rate (min^−1^)^+^	Score 0	other	31.8%	50.0%
	Score 1	-	-	-
	Score 2	5–6	68.2%	50.0%
	M (SD)		119 (12)	121 (7)

Time to first heart rhythm analysis (s)	Score 0	>138	16.1%	6.3%
	Score 1	121–138	3.3%	0.0%
	Score 2	<=120	80.6%	93.7%
	M (SD)		98 (53)	74 (30)

Time to the first shock (s)*	Score 0	>138	25.8%	18.8%
	Score 1	121–138	9.7%	9.3%
	Score 2	<=120	64.5%	71.9%
	M (SD)		135 (82)	111 (67)

Expected number of person providing chest compression changes (in relation to scenario length)	Score 0		38.7%	12.5%
	Score 1		41.9%	46.9%
	Score 2		19.4%	40.6%
	M (SD)		NA	NA

Deviation from person providing chest compression change algorithm (s)	Score 0	>36	54.8%	31.3%
	Score 1	19–36	22.6%	18.7%
	Score 2	<=18	22.6%	50.0%
	M (SD)		59 (68)	29 (28)

Expected number of adrenaline administrations (in relation to scenario length)	Score 0		3.2%	3.1%
	Score 1		9.7%	15.6%
	Score 2		87.1%	81.3%
	M (SD)		NA	NA

Deviation from adrenaline algorithm (3–5 min)	Score 0	other	74.2%	59.4%
	Score 1	-	-	-
	Score 2	0	25.8%	40.6%
	M (SD)		51 (70)	17 (23)

Expected number of heart rhythm checks (in relation to scenario length)	Score 0		9.7%	0.0%
	Score 1		9.7%	3.1%
	Score 2		80.6%	96.9%
	M (SD)		NA	NA

Deviation from heart rhythm algorithm (s)	Score 0	>36	19.4%	6.3%
	Score 1	19–36	45.2%	12.5%
	Score 2	<=18	35.5%	81.3%
	M (SD)		31 (31)	12 (11)

Amiodarone administration	Score 0	None	25.8%	18.8%
	Score 1	Given	0.0%	0.0%
	Score 2	3^rd^shock	74.2%	81.3%
	M (SD)		NA	NA

+due to technical errors, the *SimPad®* data were not available for nine participants in the No App group and five participants in the CA App group; *one participant in the CA App group did not defibrillate the patient.

Based on the effect of a previous version of the application on the no-flow fraction,^16^ an a priori power analysis with 1–β = 0.08, α = 0.05, two-sided Mann-Whitney-U test, and a large effect (d = 0.8) resulted in a sample size of 2 × 33 teams. Data were analysed using independent t-tests or Mann-Whitney-U tests if the dependent variable was not normally distributed based on a Kolmogorov-Smirnov test. Alpha was set at 0.05.

## Results

We collected data of 67 teams. Four teams were excluded from the CPR performance score analysis. In two cases, the technical issues with the simulation manikin occurred and the scenario was not finished. In two further cases, participants chose wrong treatments due to erroneous interpretation of the ECG lead and hence misjudging the underlying heart rhythm (asystole instead of ventricular fibrillation). In both cases, the scenario deviated considerably from the scenario script and were not finished, making it impossible to calculate a CPR performance score.

For the remaining 63 teams, the demographic variables (gender, age, work experience in years) and scenario length of the final sample (CA App: n = 32, No App: n = 31) showed no significant differences (Table 1). One team in the CA App group never defibrillated the patient despite a correct diagnosis of the heart rhythm (ventricular fibrillation) but finished the scenario according to the scenario script. Finally, the simulator’s software failed to record the chest compression depth and rate of 13 teams. The CPR performance score of these teams was calculated based on the remaining ten variables. Note that removing the variables chest compression depth and rate for all participants did not change the result pattern.

Considering application use in the CA App group, 26 physicians kept the tablet always or almost always in their hands. The other six physicians put the tablet on the ground beside them but still used the application. The log file of the application showed on average 1.90 physical interactions (i.e., pressing a button of the application) per minute (SD = 0.43, range 1.11–3.10). This data shows that the physicians in the CA App group used the application.

**Fig. 2:**
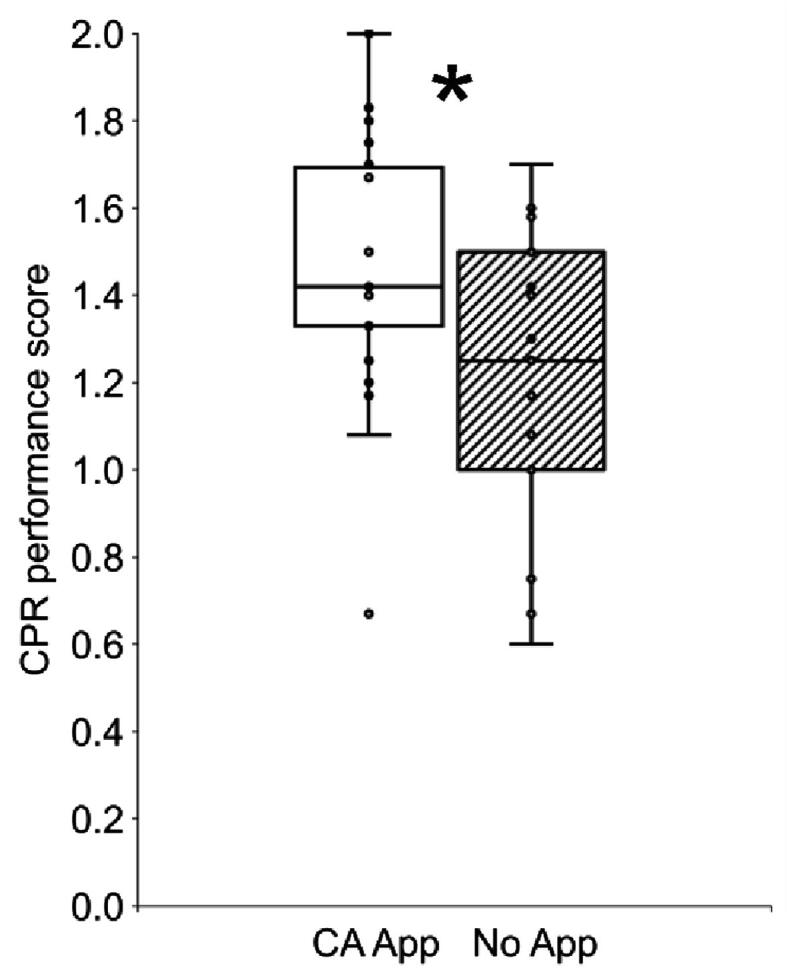
Box plots for cardiopulmonary resuscitation (CPR) performance scores separated by cognitive aid application (CA App) vs. no application (No App) groups. *t-test p < 0.05.

The CPR performance score was significantly higher in the CA App group (M = 1.47, SD = 0.29) compared to the No App group (M = 1.24, SD = 0.29, mean difference = 0.23, 95 %CI = 0.08–0.38, p = 0.002, d = 0.83, Fig. 2). The CA App group showed a descriptive better performance than the No App group in every variable and similar performance in average chest compression rate and expected number of adrenalin administrations (Table 2, rows with Scores). The same pattern was observed when considering the means of the variables (Table 2, rows with M (SD)). Finally, in an exploratory analysis, we correlated the CPR performance score and the average application use per minute and observed a significant positive correlation of r = 0.355 (p = 0.046, 95% CI = −0.101 to 0.674).

For the physicians, the analysis of the NASA TLX scores indicated significantly lower mental demand, physical demand, and effort for the CA App group than the No App group (Table 3). For the nurses, the analysis of the NASA TLX scores indicated significantly lower mental demand for the CA App group compared to the No App group. The nurses in the No App group subjectively perceived their overall performance as significantly more successful than the CA App nurses.

**Table 3:** NASA TLX results separated by cognitive aid application (CA App) and the no application (No App) groups. Data are presented as median (IQR). Statistical test are Mann-Whitney-U tests and effect sizes are rank biserial correlations. Note that low scores indicate less workload or better perceived performance.

NASA TLX Scale		CA App (n = 32 teams)	No App (n = 31 teams)	p value and effect size
Mental	Physician	13.00 (5.75)	15.00 (4.00)	p = 0.040, r = 0.300
	Nurse	10.00 (8.50)	12.00 (8.00)	p = 0.044, r = 0.298
Physical	Physician	3.50 (3.00)	5.00 (4.00)	p = 0.018, r = 0.345
	Nurse	9.50 (10.00)	9.00 (7.50)	p = 0.491, r = -0.102
Temporal	Physician	10.00 (6.50)	8.00 (8.50)	p = 0.923, r = -0.015
	Nurse	6.00 (6.25)	7.00 (8.50)	p = 0.436, r = 0.115
Performance	Physician	7.00 (5.50)	7.00 (4.00)	p = 0.837, r = 0.031
	Nurse	8.50 (6.00)	6.00 (4.50)	p = 0.032, r = 0.314
Effort	Physician	12.00 (6.25)	15.00 (4.00)	p = 0.046, r = 0.292
	Nurse	9.00 (6.25)	11.00 (7.00)	p = 0.164, r = 0.205
Frustration	Physician	7.00 (8.25)	7.00 (9.50)	p = 0.331, r = -0.143
	Nurse	5.50 (8.00)	4.00 (8.50)	p = 0.841, r = -0.030
TLX Raw	Physician	8.92 (4.93)	10.50 (3.83)	p = 0.277, r = 0.160
	Nurse	8.08 (3.99)	8.00 (5.25)	p = 0.650, r = 0.068

## Discussion

This randomized study including 63 teams demonstrated significantly improved CPR performance when using the cognitive aid application compared to no application use. Furthermore, more frequent application use was associated with improved CPR performance score. Adding to previous research on in-hospital resuscitations,8., 9., 11. we showed that cognitive aids can improve guideline-conforming CPR in relation to the timing of interventions. We demonstrated that this also holds true for well-trained and specialized emergency teams.

The benefits of cognitive aids have frequently been attributed to reduced workload^10^ due to the aid possibly functioning as a memory aid. However, this explanation has been challenged^15^ and is not empirically supported.^13^ Based on the present data, we speculate that the reduced mental workload was the result of fewer attention shifts between the different threads of CPR coordination because the CA App provided the backbone for a structured CPR coordination. The reduced physical demand is likely because the team leaders were holding the tablet in their hand and therefore did not get engaged in manual tasks. This interpretation is supported by previous work that showed that increased cognitive aid use is associated with less physical hands-on involvement.^15^

Our approach to the design of the cognitive aid application included a user-centred design process. Poor cognitive aid design is one of the barriers to emergency aid use.22., 23. Similar to others,24., 25. we think it is important to consider the context in which the aids are used, the specific users, the potential technology, and the cognitive processes that the cognitive aid should support in the design of cognitive aids. Including potential users in the design process is likely to produce a better aid and may also improve the acceptance of the aid which can be considered as another barrier to emergency aid use.

Two participants’ initial diagnoses of the heart rhythm and the resulting therapeutic management were not correct (asystole instead of ventricular fibrillation), and one participant correctly recognized the ventricular fibrillation but did not defibrillate the patient. All three participants were in the CA App group; however, it is unlikely that these errors were caused by the use of the CA App because the application did not provide any assistance or suggestions for ECG signal interpretation or the therapeutic management. This result highlights, however, that cognitive aids should provide support for the timing and coordination of events in addition to supporting diagnoses and the resulting therapeutic management decisions. Future electronic CPR aids could receive and interpret the ECG signal (like automated external defibrillators) to support the diagnoses and incorporate algorithms from existing emergency manuals^26^ to support therapeutic management decisions.

The study has limitations. First, the study was conducted in a simulated environment using a single scenario. It is uncertain whether the performance benefits translate to real CPR events or to other scenarios. In relation to other scenarios, however, we observed a reduced no-flow fraction in two other simulation-based scenarios in a study with a previous version of the application (acute pulmonary embolism with rapid deterioration; consecutive cardiac arrest and acute cardiac arrest resulting from hyperkalemia).^16^ Second, although CPR performance score variables were derived from current guidelines,^20^ the scoring system and process has not been formally validated such as, for example, a scoring checklist for advanced cardiac life support certification.^27^ However, all variables all variables were derived from guidelines.^20^ We therefore consider our variables as a valid selection to assess the coordination of advanced CPR performance. Furthermore, the interrater agreement analyses indicated good reliability. Third, due to the fact that the CA App was visible on the videos when the performance score variables were coded, the coders were not blinded to the intervention. However, there was little need to interpret a variable, because all variables were time-stamped data (e.g., time to first shock) or discrete actions (e.g., for amiodarone administration: “none” or “given any time” or “given after 3rd shock”) and interrater agreement was excellent. Fourth, we focused on guideline-conforming CPR as the primary outcome. We need to make the assumption that guideline-conforming CPR results in improved patient outcome; however, this assumption is supported in the literature.^3^

Overall, this simulation-based study demonstrated that cognitive aids can be designed to support healthcare staff beyond supporting staff as a memory aid by prompting steps or guiding them through an algorithm and improving the coordination of CPR events. Designing a cognitive aid as an attentional aid seems to improve CPR event coordination even in well-trained emergency teams. Future applications should attempt to merge the attentional and memory aid functions of cognitive aids for support of in-hospital CPR.

## CRediT authorship contribution statement

**T. Grundgeiger:** Conceptualization, Methodology, Formal analysis, Writing - original draft, Visualization. **F. Hahn:** Investigation, Writing - review & editing. **T. Wurmb:** Conceptualization, Methodology, Writing - review & editing. **P. Meybohm:** Resources, Writing - review & editing. **O. Happel:** Conceptualization, Methodology, Validation, Investigation, Writing - review & editing.

## Declaration of Competing Interest

The authors declare that they have no known competing financial interests or personal relationships that could have appeared to influence the work reported in this paper.
